# Circulating microRNAs in post myocardial infarction remodeling: toward precision cardiology

**DOI:** 10.1097/MS9.0000000000004468

**Published:** 2025-12-03

**Authors:** Muhammad Khizar, Muhammad Zaib, Muhammad Bilal, Hasibullah Aminpoor, Hasiba Karimi

**Affiliations:** a Faculty of Medicine, Georgian American University Manifest Medical Research Co, Tbilisi, Georgia; b Faculty of Medicine, Shahida Islam Medical and Dental College, Lodhran, Punjab, Pakistan; c Faculty of Medicine, Kabul University of Medical Sciences “Abu Ali Ibn Sina”, Kabul, Afghanistan; d Faculty of Medicine, Bezmialem Vakif University, Istanbul, Turkey

**Keywords:** biomarkers, circulating microRNAs, fibrosis, myocardial infarction, precision cardiology, ventricular remodeling

## Abstract

Post-myocardial infarction (MI) ventricular remodeling is a major cause of heart failure, despite advances in revascularization and pharmacotherapy. Circulating microRNAs (miRNAs) are emerging as biomarkers for risk stratification and therapeutic targeting. This letter reviews their role in apoptosis, inflammation, and fibrosis, highlights studies from Europe, Asia, and North America, and discusses technological advances, including exosome-based miRNA panels and machine learning models. Limitations include analytical variability, limited geographic representation, and lack of integration into clinical care. Recommendations include standardizing methods, conducting large international studies, and developing actionable miRNA algorithms. Integrating miRNA profiling into post-MI care has the potential to improve risk assessment, personalize therapy, and reduce the burden of heart failure.

*To the Editor,*

The transition from acute myocardial infarction (MI) to adverse left ventricular remodeling and ultimately heart failure remains a central challenge in cardiovascular medicine. As survival after primary percutaneous coronary intervention improves globally, attention increasingly turns to identifying patients at risk of post-infarction structural changes and intervening early. Circulating microRNAs (miRNAs) have emerged as promising biomarkers that combine mechanistic insight with clinical utility, offering a path toward precision cardiology.

In the early phase after MI, cardiomyocyte death triggers an inflammatory response, fibroblast activation and extracellular matrix deposition. These processes alter ventricular geometry, leading to dilation and impaired function. Fibrosis, mediated by TGF-β/Smad and other signaling pathways, is a major driver of this maladaptive remodeling^[1]^. miRNAs, small non-coding RNAs that regulate gene expression after transcription, modulate apoptosis, hypertrophy, inflammation, and fibrosis in the injured myocardium^[2]^. miR-21, miR-29b, and miR-133a have been specifically linked to fibroblast activation and collagen deposition^[3]^.

Clinically, circulating miRNAs are attractive because they are stable in blood, reflect tissue specific injury, and can rise earlier than conventional biomarkers such as troponin or natriuretic peptides^[4]^. In a Spanish study, plasma miR-1254 predicted adverse ventricular remodeling at six months in STEMI patients, independent of infarct size and imaging results^[5]^. Studies from China and the United States have expanded evidence for miRNAs in acute coronary syndromes and post-MI remodeling^[6]^. These results suggest that incorporating circulating miRNA profiling into routine post-MI care in Europe, Asia, and North America could improve risk stratification, guide therapy, and reduce progression to heart failure.

Technological advances have improved miRNA detection and interpretation. Exosome-derived miRNA isolation, small RNA sequencing, and machine learning allow multi-miRNA panels to be developed rather than relying on single markers^[2]^. For example, platelet-derived miRNAs regulate the inflammatory-to-fibrotic transition after myocardial ischemia-reperfusion injury, highlighting potential therapeutic targets^[7]^. Therapeutically, modulating miRNA expression, such as inhibiting pro-fibrotic miR-21 or restoring anti-fibrotic miR-29 family members, is under investigation, supporting a future where miRNA profiling informs both diagnosis and treatment^[8]^.

Despite these advances, miRNA application in post-MI remodeling remains limited. First, pre-analytic factors, including timing of sampling, anticoagulant use, and storage conditions, vary across centers, reducing reliability^[4]^. Second, most studies are small, single-center, and concentrated in high-income countries. Data from middle-income countries such as India, South Africa, and Brazil are limited, affecting generalizability^[3]^. Third, while many miRNAs are associated with remodeling, few studies have demonstrated that adding miRNA panels to existing clinical or imaging models improves decision-making or cost-effectiveness. Achieving impact will require large international studies with diverse populations, standard methods, and miRNA-guided decision algorithms.

In conclusion, circulating miRNAs are promising tools for precision cardiology after MI, offering insight into mechanisms, risk stratification, and potential therapeutic targets. To translate this into practice, we recommend standardizing sampling and assay methods, including under represented regions in research, validating multi-miRNA panels in large cohorts, and integrating miRNA guided stratification into routine care. With these steps, miRNA-based risk assessment and targeted therapy may become standard practice and reduce the global burden of post-infarction heart failure.

Our work is in line with the TITAN Guidelines on the need for transparency in AI use in healthcare^[9]^.

## Data Availability

Not applicable.
